# A Systematic Review of Ebstein’s Anomaly with Left Ventricular Noncompaction

**DOI:** 10.3390/jcdd9040115

**Published:** 2022-04-13

**Authors:** Suma K. Thareja, Michele A. Frommelt, Joy Lincoln, John W. Lough, Michael E. Mitchell, Aoy Tomita-Mitchell

**Affiliations:** 1 Department of Cell Biology, Neurobiology, and Anatomy, Medical College of Wisconsin, Milwaukee, WI 53226, USA; sthareja@mcw.edu (S.K.T.); jlough@mcw.edu (J.W.L.); 2 Department of Surgery, Division of Congenital Heart Surgery, Medical College of Wisconsin, Milwaukee, WI 53226, USA; memitchell@mcw.edu; 3 Department of Pediatrics, Division of Pediatric Cardiology, Children’s Wisconsin, Milwaukee, WI 53226, USA; mafrom@mcw.edu (M.A.F.); jlincoln@mcw.edu (J.L.); 4 Herma Heart Institute, Children’s Wisconsin, Milwaukee, WI 53226, USA

**Keywords:** Ebstein’s anomaly, left ventricular noncompaction, congenital heart disease, cardiomyopathy, genetic etiology

## Abstract

Traditional definitions of Ebstein’s anomaly (EA) and left ventricular noncompaction (LVNC), two rare congenital heart defects (CHDs), confine disease to either the right or left heart, respectively. Around 15–29% of patients with EA, which has a prevalence of 1 in 20,000 live births, commonly manifest with LVNC. While individual EA or LVNC literature is extensive, relatively little discussion is devoted to the joint appearance of EA and LVNC (EA/LVNC), which poses a higher risk of poor clinical outcomes. We queried PubMed, Medline, and Web of Science for all peer-reviewed publications from inception to February 2022 that discuss EA/LVNC and found 58 unique articles written in English. Here, we summarize and extrapolate commonalities in clinical and genetic understanding of EA/LVNC to date. We additionally postulate involvement of shared developmental pathways that may lead to this combined disease. Anatomical variation in EA/LVNC encompasses characteristics of both CHDs, including tricuspid valve displacement, right heart dilatation, and left ventricular trabeculation, and dictates clinical presentation in both age and severity. Disease treatment is non-specific, ranging from symptomatic management to invasive surgery. Apart from a few variant associations, mainly in sarcomeric genes *MYH7* and *TPM1*, the genetic etiology and pathogenesis of EA/LVNC remain largely unknown.

## 1. Introduction

Congenital heart defects (CHDs) are cardiogenic malformations present at or before birth that account for a high burden of neonatal morbidity and mortality in the developed world. As improvements in cardiac medicine enable patients with CHDs to live longer, there remains an unmet need for tailored medical care and treatment. Here, we provide a brief overview of two rare CHDs—Ebstein’s anomaly (EA) and left ventricular noncompaction (LVNC)—and thoroughly review publications that discuss combined EA/LVNC.

### 1.1. Ebstein’s Anomaly (EA)

Reported EA prevalence ranges from one in 200,000 to, more commonly, one in 20,000 live births, and accounts for less than one percent of CHD cases [1,2,3,4]. Improved patient health outcomes, advanced diagnostic imaging, and genomic technology likely contribute to this epidemiological shift [4].

EA mainly affects the tricuspid valve and right ventricle. Specific EA characteristics include the attachment of posterior and septal leaflets to the ventricular septum, apical displacement of the valve, redundant and fenestrated anterior leaflet, atrialization of the portion of right ventricle above the valve, and dilation of the right ventricle and atrioventricular junction [1,5,6].

Significant EA pathology originates from the right heart dilation, and consequently, over two-thirds of EA patients display enlargement of the right heart, including the atrialized inlet portion of the right ventricle and often the functional right ventricular apex and outflow tract [1]. Pronounced dilation also narrows the left ventricular chamber (visibly shaped as a crescent on an echocardiogram, or ECHO) and may lead to heart failure from left ventricular outflow tract obstruction [1,7].

Clinical heterogeneity of EA patients ranges in anatomic and physiologic severity, and as such, treatment from medication to valve repair is highly individualized [8,9]. Fetal presentation or severity of clinical symptoms increases risk of mortality [10]. Common EA-associated cardiac comorbidities are atrial septal defect (ASD), Wolff–Parkinson–White (WPW) syndrome, and right ventricular outflow tract obstruction [11]. Neonates born on the verge of viability commonly exhibit symptoms of heart failure (e.g., poor feeding, failure to thrive, and dyspnea) [1]. Survival to adulthood is variable and dependent on clinical severity; thus, EA prevalence in adults is not thoroughly enumerated.

Categorization of EA as mild, moderate, or severe is based on varied classification systems: (1) Carpentier et al. [12] assess freedom of valve leaflet movement and severity of outflow tract obstruction; (2) Celermajer et al. [10] grade severity based on volumetric chamber measurements from ECHO [10]; and Dearani et al. [13] review both the cardiac morphology on ECHO and leaflet anatomy from surgical observation.

### 1.2. Left Ventricular Noncompaction (LVNC)

EA also commonly manifests with left ventricular noncompaction (LVNC), a cardiomyopathy of ventricular myocardium that does not transit from a weak and “spongy” stage to a compacted state during development [14,15]. Morphological characteristics include abnormal trabeculations and deep intra-trabecular recesses adjacent to the endothelium that resemble the morphology of the right ventricle.

Common LVNC-associated cardiac comorbidities include ventricular septal defect (VSD), ASD, and patent ductus arteriosus (PDA). One pathologic predictor of LVNC is a greater base–apex strain, resulting from longitudinal deformation [16].

Prevalence of LVNC is even more variable than EA, and diagnosis may be missed in patients with asymptomatic clinical presentation or absent imaging. Similar to EA, early LVNC presentation in infancy increases the risk of mortality [17]. Often, the left ventricle is dilated, or hypertrophic, and severe cases may result in heart failure from either or both systolic and diastolic dysfunction [15]. Men are more likely to present with noncompaction (56–82% of cases) than women [18].

At least eight different LVNC phenotypes that range from benign to restrictive pathology with variable clinical associations such as ventricular dilation, arrhythmias, and systolic dysfunction exist [15]. Three common criteria for a diagnosis of LVNC include a ratio of the length of the compacted wall (x) to the trabeculated wall (y) being less than 0.5 (i.e., *x*/*y* < 0.5) on short-axis ECHO [19], a noncompacted to compacted ratio (NC:C) greater than 1.4 in children or 2 in adults, also on short-axis ECHO [20], and a NC:C ratio greater than 2.3 on long-axis cardiac MRI [21].

### 1.3. Ebstein’s Anomaly with Left Ventricular Noncompaction (EA/LVNC)

**Figure 1:**
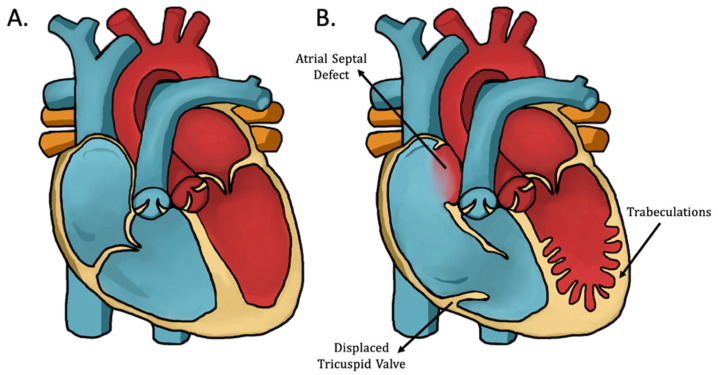
Illustration of EA/LVNC morphology: (**A**) normal heart; (**B**) morphological defects seen in EA/LVNC. Common characteristics depicted here are the apical displacement of the tricuspid valve, dilation of the right atrioventricular junction, trabeculated left ventricular myocardium, and an atrial septal defect (a common comorbidity of EA/LVNC). EA/LVNC; Ebstein’s anomaly with left ventricular noncompaction.

Illustrated in Figure 1 are the morphological alterations of combined EA and LVNC (EA/LVNC), which is a specific category of EA patients that was first described by Attenhofer Jost et al. in 2004 [22]. The spectrum of phenotypes for both CHDs underscores both the putative genetic etiology and the complexity of the G × E interaction during disease development.

## 2. Methods

**Figure 2:**
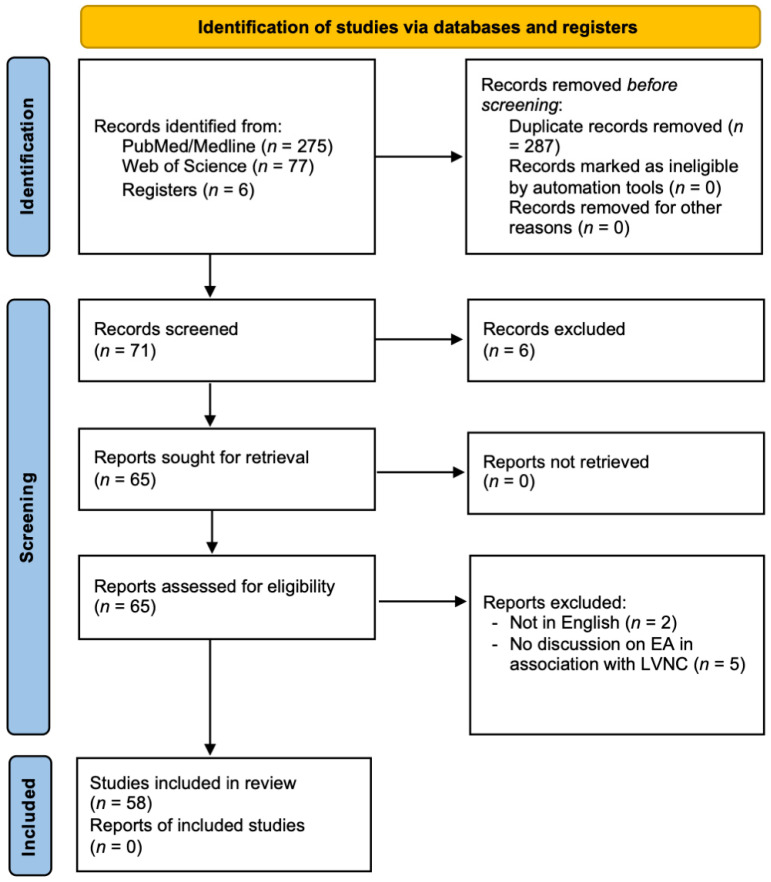
PRISMA 2020 flow diagram [23,24] for this systematic review on EA/LVNC.

We followed the systematic review guidelines of the Preferred Reporting Items for Systematic Reviews and Meta-Analyses (PRISMA) [23,24] and identified all peer-reviewed publications from inception to February 2022 that considered EA/LVNC (Figure 2). We accessed PubMed, Medline, and Web of Science with the search terms “(Ebstein’s anomaly) and (left ventricular noncompaction)”, “(Ebstein’s anomaly) AND (left ventricular non-compaction)”, “(Ebstein’s anomaly) AND (LVNC)”, “(Ebstein’s anomaly) AND (left ventricular hypertrabeculation)”, “(Ebstein’s anomaly) AND (noncompaction)”, and “(Ebstein’s malformation) AND (noncompaction)” for relevant articles. We also queried clinical trials or studies noted on ClinicalTrials.gov and clinicaltrialsregister.eu. We then tabulated and created tables of EA/LVNC clinical signs, imaging modalities, treatments, clinical trials, and genetic associations. To eliminate risk of bias while S.K.T. independently reviewed all articles, we only excluded publications that were not written in English or did not consider combined EA/LVNC.

## 3. Results

### 3.1. We Identified 58 Peer-Reviewed Publications That Discuss EA/LVNC

As presented in the PRISMA flow diagram (Figure 2) [23,24], we identified a total of 358 papers from inception to February 2022 from the use of the search terms applied to manuscripts. Of these, 287 were redundant. Among the 71 unique articles, we excluded 6 following title/abstract screening [25,26,27,28,29,30]. We retrieved 65 full-text articles and further excluded 2 articles that were not written in English [31,32] and 5 with no direct focus on EA/LVNC in association with each other or on a morphologically similar spectrum [1,5,6,33,34]. This resulted in 58 unique articles published between 2004 and 2022, including sporadic case reports [22,35,36,37,38,39,40,41,42,43,44,45,46,47,48,49,50,51,52,53,54,55,56,57,58,59,60,61,62,63,64,65,66,67], familial case reports [68,69,70,71,72,73,74,75], large-scale clinical or genetic studies [7,17,18,76,77,78,79,80,81,82,83], and reviews on related clinical manifestations [84,85,86,87,88].

### 3.2. EA/LVNC Patients Present with Clinical Signs of Both CHDs

Dual EA/LVNC patients present with clinical characteristics of both myocardial disorders (Figure 1B) [22,76,77]. As EA commonly manifests with LVNC, the incidence of EA with LNVC is estimated to be between 15% and 29% [78,79,81,82] and much lower (<5%) when only adult patients are considered [80]. There appears to be no clear increased prevalence, risk, or differing phenotypes of EA or LVNC associated with race.

Table 1 summarizes common clinical signs of EA/LVNC based on age. Childhood presentation is associated with signs of heart failure, while late adulthood is associated with asymptomatic or incidental finding. All other age ranges in case reports display variable clinical signs, including exertional dyspnea, chest pain, and arrhythmia. Among all sporadic and familial case reports, two most common clinical signs are exertional dyspnea and systolic murmur from tricuspid regurgitation (Table 2). Larger-scale studies of EA, LVNC, or EA/LVNC are outlined in Table 3.

Patients born with both EA and LVNC and diagnosed in infancy have a higher risk of poor outcomes such as heart failure and sudden cardiac death and manifest with anatomical variations that pose an additional surgical challenge [7,55,82,83]. Thus, a decreased incidence of dual EA/LVNC presentation in adulthood appears to correlate with increased morbidity. Similar to individual EA or LVNC, septal defects (ASD or VSD) are the most common comorbidities of EA/LVNC [22,44,47,63,69,70].

In cases of combined EA/LVNC, typically one of the two conditions is present in the relatively mild state. Some case reports describe patients initially diagnosed by EA and a later diagnosis of LVNC following an ischemic attack or unexplained clinical phenomena that prompted additional imaging [38,62,66]. Examples also exist where a patient will exhibit EA, LVNC, and other cardiac abnormalities [36,52], yet typically one of the conditions presents latently or only intermittently. In certain cases, significant remodeling of the heart is noted, presumably from acclimatization to an anatomical and hence physiological challenge [51]. The converse conclusion may be that in most instances where morphological features of both CHDs appear—or would have appeared if the patient survived—the ontological pathways that react to the dual challenge were not available or failed. Clinicians and researchers studying EA/LVNC postulate that there may be some link between both diseases as evidenced by a strong, if not complete, genetic component, and thus, an abnormal pathology would presumably exist in those affected from birth.

### 3.3. EA/LVNC Patients Commonly Present with Arrhythmia

A clinical association of arrhythmia for patients with EA/LVNC originated as early as 1978. Monibi et al. evaluated 17 newborns with EA and found that 13 presented with high normal or elevated hematocrit without cyanosis, and 9 of 13 who had LV catheterization and cineangiocardiography displayed abnormal contraction with unusually elongated and distorted long axis and irregularity of septal and lateral walls [89]. Of the 13 patients, 12 had abnormal contraction, hypokinesis, or dyskinesis [89].

Two decades hence, cineangiography of 26 adults with EA identified that 16 presented with abnormal LV contraction (hypokinesia in 10, dyskinesia in 6, and premature diastolic distention of the anterobasal wall in 8) [90].

**Table 3:** Clinical Studies that discuss EA/LVNC.

Study	Published	Focus	Center	Subjects	Gender	EA/LVNC	Results
Attenhofer Jost et al. [78]	2005	Clinical study of EA	Mayo Clinic Rochester, MN, USA	106 patients aged 0–52 years.	39.7% M 60.3% F	19 EA patients with LVNC (18%)	39% of EA patients exhibited LV abnormalities including LVNC, bicuspid aortic valve, VSD, and mitral valve abnormality Decreased trend of the ECG QRS axis mean in EA/LVNC (12°) compared with all patients (36°)
El-Menyar et al. [18]	2007	Clinical study of LVNC in Qatar	Hamad General Hospital Doha, Qatar	12 patients aged 0–37 years.	33% M 67% F	1 LVNC patient with EA	25% mortality rate 50% LVNC patients associated with CHD (VSD, PS, EA, coarctation) Poor prognosis associated with biventricular noncompaction
Reemtsen et al. [7]	2007	Clinical study of EA	Children’s Hospital Los Angeles Los Angeles, CA, USA	12 patients aged 0–17 years.	58% M 42% F	All neonates with EA had severe LV dysfunction	12 of 16 patients aged 0–17 years. survived neonatal RV exclusion surgery 11 of 12 survivors received a bidirectional Glenn shunt and 6 were completely palliated with a Fontan Prior to Glenn and Fontan, patients had a decreased CT ratio, Great Ormond Street ratio, RV/LV ratio, septal impingement from a/b approaching ratio, and shortening fraction; all these parameters improved following Glenn and Fontan
Tsai et al. [17]	2009	Clinical study of LVNC	Riley Hospital for Children Indianapolis, IN, USA	46 patients	50% M 50% F	5 LVNC patients with EA	54% heart failure 52% decreased LV ejection fraction 78% associated cardiac defects (ASD, VSD, PDA, EA) 80% ECG abnormalities 20% mortality rate not correlated to ejection fraction, morphological defect, or arrhythmia
Stähli et al. [79]	2013	Clinical study of LVNC	University Children’s Hospital Zurich, Switzerland	202 patients	79% M 21% F	6 EA patients with LVNC (15%)	24% associated cardiac defects (aortic valve abnormalities, EA, TOF, DORV)
Pignatelli et al. [82]	2014	Clinical study of EA or EA/LVNC	Texas Children’s Hospital Houston, TX, USA	61 infants		51 infants with EA 10 EA infants with LVNC (16.4%)	EA/LVNC cohort trended in earlier detection (9/10 patients were diagnosed at birth) and a higher risk of adverse outcomes (progressive LV dysfunction) than patients with EA alone
Kumor et al. [80]	2018	Clinical study of EA	Institute of Cardiology Warsaw, Poland	84 patients aged 16–71 years.	41% M 59% F	4 EA patients with LVNC (4.8%)	ASD type II (27.3%) and WPW syndrome (10.7%) were common in EA patients EA/LVNC patients alone in this cohort suffered cardiac arrest or ventricular arrhythmia
Hirono et al. [83]	2020	Clinical and genetic study of LVNC	University of Toyama Toyama, Japan;	53 Japanese probands aged 1–14 years.	47% M 53% F	7 LVNC patients with EA	LVNC/CHD patients had lower ejection fractions, thickened trabeculations, and worse prognosis when compared with age-matched patients with ventricular septal defects Heart failure, LV ejection fraction of <24%, LV end-diastolic diameter z-score of >8.56, and the LV NC:C ratio >8.33 at the last visit were risk factors for survival 30 genetic variants in 28 patients with LVNC and CHD in the genes (50% sarcomeric): *MYH7*, *TPM1*, *ACTC1*, *ANK2*, *COL4A1*, *DAAM1*, *DSG2*, *DSP*, *FGF16*, *FGFR2*, *HCN4*, *JUP*, *MYBPC3*, *MYH6*, *MYL2*, *PKP2*, *PRDM16*, *RYR2*, and *TAZ*
Marques et al. [81]	2020	CHD study of postnatal heart specimens	Heart Institute (InCor), University of Sao Paulo Medical School, Brazil	259 postnatal hearts with 87.3% aged less than 18 years.	49% M 51% F	23 patients with EA	Prevalence of LVNC in EA patients was either 28.6%, 9.5%, or 0% based on LVNC diagnostic criteria using Chin’s, [91] Jenni’s [20] or Petersen’s [21] approaches VSD hearts presented significantly higher with LVNC regardless of the three methods of LVNC diagnosis

CHD: Congenital Heart Defect, DORV: Double Outlet Right Ventricle, EA: Ebstein’s Anomaly, ECG: Electrocardiogram, LV: Left Ventricle, LVNC: Left Ventricular Noncompaction, PDA: Patent Ductus Arteriosus, PS: Pulmonary Stenosis, RV: Right Ventricle, TOF: Tetralogy of Fallot, VSD: Ventricular Septal Defect.

Among 106 EA patients studied between 2001 and 2003 at the Mayo Clinic, there were 76 severe cases, 19 (18%) presented with LVNC, and 22 (21%) with WPW syndrome [78]. While no correlation existed between anatomical EA severity with the degree of LVNC, the QRS axis mean, which depicts ventricular depolarization on an electrocardiogram (ECG), showed a marginally significant difference (12° mean in EA/LVNC patients when compared with the 36° of all patients) [78].

In a study of 12 survivors among 16 neonates with EA who underwent right ventricular exclusion surgery, shortening fractions were found to be significantly lower prior to follow-up Glenn and Fontan procedures [7]. In our review, arrhythmia is a common clinical sign of EA/LVNC that appears second only to exertional dyspnea.

### 3.4. ECHO Is Preferred for EA Diagnosis While Cardiac MRI Is Preferred for LVNC

All imaging modalities used for diagnosis and EA/LVNC characterization are noted in Table 2. Imaging via ECHO is preferred for both CHDs, as it is noninvasive and cost-friendly [92]. However, LVNC diagnosis using ECHO is controversial because of a perceived arbitrary interpretation in imaging [15]. Cardiac MRI is thus recommended for diagnosing LVNC [45,56]. For quantitative assessment of volumetric and functional fractions, a cardiac CT may be used. Additional methods that offer further insight but less specificity and sensitivity for EA and LVNC include ECG, pulse oximetry, and exercise tests. In our review, we found most studies presented imaging and figures from 2-D ECHO, color flow doppler ECHO, cardiac MRI, and an ECG (Table 2).

### 3.5. EA/LVNC Treatment Is Non-Specific and Heterogenous

EA/LVNC treatment (Table 2) is nonspecific and ranges from symptomatic management to surgical valve repair [93,94]. Commonly, treatment included medication for heart failure or anticoagulation. Mechanical ventilation was common for earlier or severe presentation. Surgical repair often comprised arterioplasty, annuloplasty, closure of septal defects, and transplantation. Arrhythmia treatment consisted of medication or devices such as an implantable cardioverter-defibrillator (ICD) or left ventricular assist device (LVAD). In one CHD and/or LVNC-associated ICD study, EA patients suffered the highest complication rate during device implant, replacement, or lead-related procedures [86]. Most case reports, however, neglected details of the treatment initiated or discussed case prognosis.

### 3.6. Most Clinical Trials of EA or LVNC Seek to Assess CHD Genetic Etiology

All clinical trial information available for EA or LVNC in the US and EU are described in Table S1 (Supplementary Materials). Most were prospective observational studies and mainly sought to stratify risk and diagnostic criteria for each CHD. Some also studied the genetic underpinnings of EA or LVNC. One active trial (NCT02914171) investigating a nonrandomized therapeutic intervention of autologous bone marrow-derived mononuclear cells to treat 10 EA patients is projected to complete soon and may offer insight into EA therapies outside of the common medication or cardiac surgical procedures. Two other trials (NCT02432092 and NCT04265040) actively recruiting patients with cardiomyopathy (including LVNC) are studying molecular genetics and all-cause mortality outcomes.

### 3.7. Sarcomeric Variants Are Implicated in the Genetic Etiology of EA/LVNC

The genetic etiology and pathogenesis of EA/LVNC are largely unknown. Studies identified clinically significant variants at a variety of loci in EA and LVNC (*MYH7* [68,69,76], *NKX2.5* [25,95], *GATA4* [96], *TAZ* [97], *TBX20* [98], and others [74,99,100,101,102]), but few have been reported in EA/LVNC (Table 4). While independent EA- [103] or LVNC-associated [83] genetic variants have been discussed in the literature, Table 4 summarizes all genetic variant associations for EA/LVNC to date.

Many of these variants are in sarcomeric or cytoskeletal proteins and have broad implications in cardiac formation, function, and conduction [71,103]. The *MYH7* locus, which encodes the β-myosin heavy chain (βMyHC), is significantly implicated in EA, LVNC, and EA/LVNC [68,69,70,75,76,77,83,87,88,104]. EA/LVNC-specific studies are detailed below.

**Table 4:** Summary of published work describing combined EA/LVNC-associated variants.

Year	Publication	Sample Size	Occurrence	Findings
2007	Budde et al. [69]	24	Familial	*MYH7* (p.R281T)
2010	Hoedemaekers et al. [104]	58	Sporadic	*MYH7* (p.L301Q)
2011	Postma et al. [76]	141	Sporadic	*MYH7*; 7 mutations (5 novel)
2014	Hirono et al. [70]	3	Familial	*MYH7* (p.M362R)
2016	Kelle et al. [50]	1	Sporadic	*TPM1* (p.D159)
2018	Nijak et al. [71]	5	Familial	*TPM1* (p.L131V)
2019	Carlston et al. [41]	1	Sporadic	*NONO* (p.N52Sfs)
2020	Hirono et al. [83]	53	Sporadic	30 genetic variants in *MYH7*, *TPM1*, *ACTC1*, *ANK2*, *COL4A1*, *DAAM1*, *DSG2*, *DSP*, *FGF16*, *FGFR2*, *HCN4*, *JUP*, *MYBPC3*, *MYH6*, *MYL2*, *PKP2*, *PRDM16*, *RYR2,* and *TAZ*
2020	Samudrala et al. [72]	17	Familial	*KLHL26* (p.R237C)
2021	Mehdi et al. [56]	1	Sporadic	Gain chromosome band 15q11.2 and 1q44
2022	Coetzer et al. [42]	1	Sporadic	*NONO* (p.M389_T400del)
2022	Tu et al. [75]	6	Familial	*MYH7* splicing variant

Following a genome-wide linkage analysis of a German familial case of LVNC with 11 affected members among 25, Budde et al. discovered the *MHY7* (p.R281T) variant. Of the 11 LVNC-affected members, 4 also presented with EA. This *MYH7* variant is suspected to prevent salt-bridge formation, destabilizing the myosin head and resulting in altered contractility [69].

Postma et al. examined the *MYH7* locus of 141 unrelated EA patients via mutation screening and detected seven distinct heterozygous mutations, of which five were novel. In total, 6 patients among the 8 who possessed these *MYH7* variants and none of the remaining 133 subjects presented with LVNC [76].

After reviewing this latter publication and a few other EA/LVNC genetic studies, Vermeer et al. concluded the association of at least nine distinct *MYH7* mutations with EA/LVNC [87]. Five of these are located within the head region of βMyHC, while the other four reside in the rod domain, which mediates filament assembly and the sliding motion of contraction. Vermeer et al. further describe a subtype of EA/LVNC resulting from *MYH7* mutations or possibly other sarcomeric variants that follow a pattern of autosomal dominant inheritance and variable penetrance [87]. This conclusion has since been supported by isolated case reports [70] and variants found in other sarcomeric proteins such as thin filament α-tropomyosin, *TPM1* (p.D159N and p.L131V) [50,71].

Most recently, in a familial case of LVNC where only the proband presented with EA/LVNC, exome sequencing of the proband and parents and subsequent Sanger sequencing of other family members identified a maternally inherited heterozygous splicing variant in *MYH7* [75].

Non-sarcomeric proteins linked to EA/LVNC include SCN5A, a sodium channel, NONO, a nonoctamer-containing POU-domain DNA-binding protein, and KLHL26, a kelch-like protein (Table 4).

Neu et al. described one EA/LVNC-affected member among four related children who presented with early cardiac arrhythmia; all four were heterozygous for the *SCN5A* (p.I230T) variant [74].

The NONO protein plays crucial roles in transcriptional regulation and RNA splicing. One frameshift and one deletion variant of NONO were discovered in EA/LVNC patients who also presented with a noncardiac syndrome [41,42].

In a separate case, one EA/LVNC patient tested negative for a panel of 17 genes, including *TPM1* and *MYH7*, but showed chromosomal gain bands in 15q11.2 and 1q44 [56].

Recently, our group identified 10 EA/LVNC-affected members out of 17 in a familial study with an autosomal dominant pattern of inheritance [72]. From exome sequencing and subsequent Sanger sequencing, we discovered that the *KLHL26* (p.R237C) variant segregated with disease. The KLHL family of proteins is known to have a role in actin binding, cytoskeletal rearrangement, and ubiquitin-mediated protein degradation.

The abundance of loci implicated in either EA or LVNC alone and combined EA/LVNC underscores the complexity and genetic uncertainty for these CHDs.

## 4. Discussion

The characterization of EA as a distinct right heart defect persisted for nearly 150 years since its discovery in 1866 by Wilhelm Ebstein. Similarly, LVNC is mainly defined as a condition of the left heart. However, in the last 10–15 years, there has been an accumulation of evidence for more than a coincidental occurrence of EA and LVNC. Because of the prevalence of right-hearted lesions in LVNC and left-hearted lesions in EA seen in this review and others [78,105], a thorough review of the whole heart is recommended during workup of EA and/or LVNC.

### 4.1. Shared Developmental Pathways of EA and LVNC

**Figure 3:**
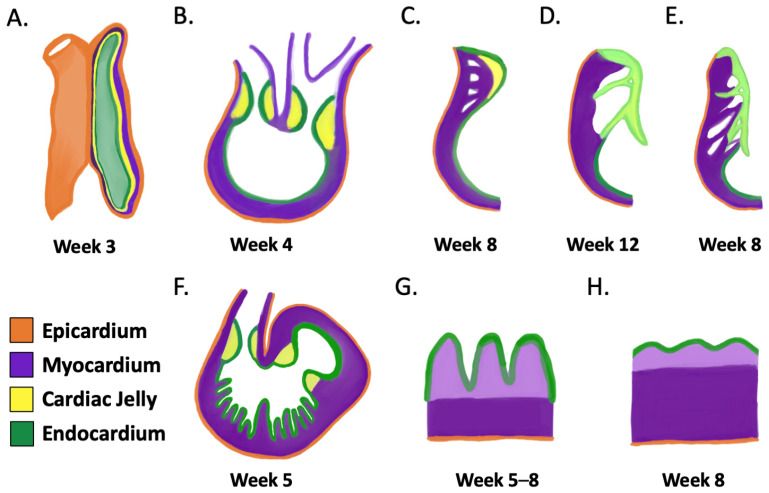
Development of the tricuspid valve and compaction of the ventricular myocardium: Cardiogenesis is initiated as (**A**) heart tube consisting of an outer epicardium that surrounds a layer of myocardial cells, with inner cardiac jelly and an endocardial cell layer; (**B**) following, the heart tube undergoes rightward looping, and endocardial cells overlying the primitive valve regions undergo endothelial-to-mesenchymal transformation and invade the cardiac jelly to form swellings termed endocardial cushions; (**C**) these cushions then remodel and elongate into leaflets/cusps and supporting structures. In the atrioventricular position (mitral, tricuspid), the leaflets separate from the myocardial wall via delamination and (**D**) become freely movable; (**E**) an example of an apically displaced valve leaflet with incomplete separation from the myocardial wall typically seen in EA; (**F**) cardiomyocyte proliferation and endocardial cell invagination form the sheet-like protrusions of the (**G**) trabecular layer seen in light purple; (**H**) later, this trabecular layer becomes compacted. Parallel signaling from TGF-β/Smad, Wnt/β-catenin, noncanonical Wnt, Notch, and Smad-independent TGF-β, BMP, and FGF pathways enable the development of both the tricuspid valve and compaction of the ventricular myocardium. Time indicated corresponds with human development.

The human heart begins to develop during the third week of human gestation [106]. The heart tube initially consists of an outer myocardial layer surround by the epicardium and an inner endocardial layer separated by cardiac jelly (extracellular matrix) (Figure 3A) [107]. Starting in week 4, a subset of overlying endocardial cells undergo endocardial-to-mesenchymal transformation (EndEMT) and invade the cardiac jelly overlying the atrioventricular canal, and the outflow tract swells to form the cardiac cushions (Figure 3B) [108,109]. In weeks 6–8, the cushions undergo extensive remodeling to form the mitral and tricuspid valves in the atrioventricular canal [110,111] (Figure 3C,D) and semi-lunar valves in the developing aorta and pulmonary trunk regions [109,111].

Cardiac fibroblasts within the developing myocardium are largely derived following epicardial-to-mesenchymal transition (EMT) of the epicardium are known, in parallel, to provide signals for development of the atrioventricular valve as well as the compact myocardium [26,112]. The processes of EndEMT and EMT are governed by multiple pathways including the TGF-β/Smad, Wnt/β-catenin, Notch, and Smad-independent TGF-β pathways [113,114]. Regulatory factors such as bone morphogenic proteins (BMPs) and fibroblast growth factors (FGFs) prevent valve hyper- or hypoplasia [107,115].

The tricuspid valve is comprised of anterior, septal, and posterior leaflets; delamination of the septal leaflet from the muscular ventricular septum [116,117] likely occurs via anoikis (integrin-mediated apoptosis) [115]. Failure of delamination is thought to result in EA, which is characterized by an apically displaced tricuspid valve with a varied degree of freedom in movement (Figure 3E).

As these events unfold, myocardial compaction of the trabeculae, which began forming in week 4, occurs in week 8 [110,118]. As the myocardium expands via cardiomyocyte proliferation, endocardial cells invaginate, forming sheet-like protrusions (Figure 3F,G) comprising the trabeculae (light purple in Figure 3G), which is theorized to facilitate exchange of oxygen and nutrients [14]. By mid-gestation, the trabeculae become compacted (Figure 3H) [8,119]. Important factors in early trabeculation are a result of signaling via endocardial and myocardial Notch, myocardial BMPs, epicardial FGFs, and both canonical and noncanonical Wnts [14].

Two LVNC investigations offered direct insight into genetic etiology. One described autosomal dominant LVNC associated with a *MIB1* (mindbomb homolog 1) variant [101]. MIB1 encodes an E3-ubiquitin ligase that promotes endocytosis of two NOTCH ligands. The second study modeled LVNC associated with a variant in transcription factor *TBX20* using induced pluripotent stem cells (iPSCs); they found decreased cellular proliferation in disease lines and showed phenotype reversal following variant correction [98].

### 4.2. A Common Etiology for EA and LVNC?

While EA/LVNC pathophysiology remains unknown, when we reflect on the considerable overlap of developmental pathways in the formation of the tricuspid valve and compacted myocardium and the genetic variants associated with both CHDs, it is unsurprising that EA commonly manifests with LVNC.

Investigating sarcomeric and cytoskeletal variants may provide evidence for arrhythmia stemming from a cellular or tissue-level contractile impediment rather than a global consequence of anatomical alterations. Aberrant contractile ability may further hinder apical constriction, depolarization, and subsequent delamination of cells during EndEMT or EMT processes [120].

## 5. Conclusions

Understanding the molecular and cellular events that unfold during the progression of disease-specific variants will provide broader insight into typical vs. aberrant cardiac development. Although clinical approaches have improved, there remains an unmet need for effective EA/LVNC treatment. Nonspecific pharmaceutical and mechanical interventions will likely remain the most common frontline treatment options for EA/LVNC until a deeper understanding of the genetic and molecular underpinnings of both CHDs becomes available.

## Figures and Tables

**Table 1:** Common clinical signs based on age of sporadic EA/LVNC case presentation.

Age of Sporadic Case Presentation	Common Clinical Signs
**Prenatal/Infancy (<1 year)** [40,42,47,56,57,60,63]	Cyanosis
**Childhood (1–12 years)** [41,50]	Heart Failure Systolic Murmur
**Adolescence (13–20 years)** [22,39,43,44,49,54,59,67] **OR** **Early Adulthood (21–40 years)** [22,37,44,45,46,51,52,53,58,61] **OR** **Middle Adulthood (41–60 years)** [35,36,38,48,55,62,64,66]	Progressive or Exertional Dyspnea Systolic Murmur Palpitations or Tachycardia Chest Pain or Discomfort Arrhythmia
**Late Adulthood (61 years+)** [65]	Progressive or Exertional Dyspnea Asymptomatic/Incidental Finding

**Table 2:** Clinical signs, imaging modalities, and interventions noted among all EA/LVNC case reports.

Clinical Signs	Imaging Modalities	Interventions/Therapies
**Arrhythmia** [35,37,43,45,51,55,61,63,65,73,74] **Asymptomatic/Incidental Finding** [36,65,66,75] **Chest Pain or Discomfort** [46,58,61,62] **Cyanosis** [47,57,74] **Exertional Dyspnea** [22,35,38,41,42,43,44,45,49,51,53,55,64,65,68,69,74] **Heart Failure** [50,73,74] **Palpitations or Tachycardia** [35,41,44,45,58,62] **Systolic Murmur** [39,40,45,48,49,54,59,66,67]	**2-D ECHO** [7,22,35,36,37,38,39,40,41,42,43,44,45,46,47,48,49,50,51,52,53,54,55,56,57,58,59,61,62,63,64,65,66,67,68,69,70,71,72,73,74,75] **3-D ECHO** [36,39,46,49,53,61,65,75] **Color Flow Doppler ECHO** [36,37,44,46,47,48,51,54,58,59,61,63,68,69,70,73,75] **Cardiac or Coronary CT Angiography** [35,46,56,62] **Cardiac MRI** [37,38,40,41,45,50,51,53,55,56,62,64,65,66,69,73] **Catheterization** [53,73] **Chest Radiograph** [7,38,43,50,51,57,58,61] **ECG** [35,36,37,41,43,44,47,48,49,51,52,54,58,61,63,65,73,74,75] **Electrophysiological Study** [35,52,73,74]	**Mechanical Ventilation** [42,50,57] **Radiofrequency Ablation** [35,58,62] **Arterioplasty/Annuloplasty** [22] **Other Cardiac Surgical Repair** [7,41,47,53,56,70] **Cardiac Transplantation** [22,44,50,69] **Atrial Septal Occluder (PFO Closure)** [38] **LVAD/BiVAD** [43,53,74] **Pacemaker/ICD** [35,55,73,74] **IABP** [53] **Inotropic Support (Epinephrine/Milirinone)** [53] **Anticoagulation (Aspirin/Warfarin/Heparin)** [35,39,43,44,45,49,52,59,65,73] **Heart Failure and Anti-arrhythmic Medications (Beta-Blockers/ACE inhibitors/Diuretics/Digoxin)** [43,44,45,46,47,51,52,57,65,73,74]

BiVAD: Biventricular Assist Device, CT: Computerized Tomography, ECG: Electrocardiogram, ECHO: Echocardiography, IABP: Intra-aortic Balloon Pump, ICD: Implantable Cardioverter-Defibrillator, LVAD: Left Ventricular Assist Device, MRI: Magnetic Resonance Imaging, PFO: Patent Foramen Ovale.

## Data Availability

Not applicable.

## Supplementary Materials

The following supporting information can be downloaded at: https://www.mdpi.com/article/10.3390/jcdd9040115/s1, Table S1: Summary of Clinical Trials on EA, LVNC, or EA/LVNC.
