# Supplementary material for: A Systematic Review of Ebstein’s Anomaly with Left Ventricular Noncompaction
Source: J Cardiovasc Dev Dis. 2022 Apr 13;9(4):115. doi: 10.3390/jcdd9040115 (PMC9031964; doi:10.3390/jcdd9040115)
Supplement: Supplementary file 1 [file jcdd-09-00115-s001.zip › jcdd-1655779-supplementary.pdf]

Table S1. Summary of Clinical Trials on EA, LVNC, or EA/LVNC.

Trial ID	Final Yr. Estimate	EA or LVNC	Study Type	Abbreviated Title	Patients	Outcome Measures	Locations
NCT00497705 *Completed*		EA	Prospective observational	Genes Causing EA	2 yrs. or older		NIH Clinical Center, U.S.A. Republican Scientific and Clinical Cardiology Center, Belarus Amosov Institute of Cardiovascular Surgery, Ukraine
NCT01907971 *Completed*	2015	EA	Prospective observational	Assessment of LV and RV Function in EA patients via different ECHO Methods	18 patients aged 11 - 80 yrs.	RV volume and function by VentriPoint Structural myocardial changes in LV (Strain) Correlate findings with clinical symptoms	University Children's Hospital, Switzerland
**NCT02914171** ***Active, not recruiting***	**2022**	**EA**	Nonrandomized intervention	**Autologous Bone Marrow Derived Mononuclear Cells for EA Treatment**	**10 patients aged 6 months-30 yrs.**	**Number and severity of adverse events and prognostic markers from start of procedure**	**Mayo Clinic, Rochester, U.S.A.**
NCT02885363 *Unknown*	2018	LVNC	Nonrandomized intervention	Prognosis of Isolated Adult LVNC	220 patients aged 18 yrs. and older	Occurrence of death, cardiac transplantation, and cardiac-related hospitalization	Assistance Publique-Hopitaux de Marseille, France
NCT01481298 *Completed*	2008	LVNC	Prospective observational	Value of CMR Derived Parameters for LVNC Diagnosis	57 patients aged 14-64 yrs.	Nonrandomized Intervention for Diagnosis of LVNC	University of Leipzig - Heart Center, Germany
NCT01470014 *Completed*	2014	LVNC	Prospective observational	Cardiac CT: Characteristics of Isolated LVNC	39 patients aged 30-90 yrs.		Zurich, Switzerland
NCT02568072 *Completed*	2016	LVNC	Prospective observational	Training-induced Increased Left Ventricular Trabeculation	120 patients aged 18-35 yrs.	NC:C ratio and peak Oxygen after exercise test	Barts Heart Centre, United Kingdom
NCT03076580 *Unknown*	2021	LVNC	Prospective observational	An Integrative-"Omics" Study of Cardiomyopathy	2000 patients of all ages	Influence of genetic variation in clinical cardiomyopathy outcomes and identifying novel biomarkers using	Beijing Institute of heart, lung and blood vessel diseases, China

				Patients for Diagnosis and Prognosis in China		proteomics, microRNA-seq, and metabolomics	
NCT03061994 *Unknown*	2020	LVNC	Prospective observational	Metabolomic Study of All-age Cardiomyopathy	1000 patients of all ages	Metabolomic profile of cardiomyopathy patients	Beijing Institute of heart, lung and blood vessel diseases, China
NCT03572569 *Unknown*	2020	LVNC	Prospective observational	Risk Stratification in Children and Adolescents with Primary Cardiomyopathy	200 patients aged up to 18 yrs.	Major cardiovascular events of pediatric patients with primary cardiomyopathy and first-degree family members	The Charité – Universitätsmedizin Berlin, Germany<br>German Heart Institute, Germany
**NCT02432092** ***Recruiting***	**2028**	**LVNC**	Prospective observational	**Pediatric Cardiomyopathy Mutation Analysis**	**300 patients of all ages**	**Molecular genetics of cardiomyopathy**	**IU School of Medicine, U.S.A.**
**NCT04265040** ***Recruiting***	**2027**	**LVNC**	Prospective observational	**DZHK TORCH-Plus is a Registry for Patients with Cardiomyopathies and Serves as Source for Cardiovascular Research Studies**	**2040 patients aged 18-80 yrs.**	**All-cause mortality**	**University Hospital Heidelberg - Clinic of Cardiology, Angiology and Pneumology, Germany**
*2019-003626-24 *Unknown status due to Brexit*		*LVNC*		*Primary Dilated Cardiomyopathy due to either MYH7 or TTN Variants.*			

Note: Trials in boldface text are ongoing as of February 2022. CMR: Cardiac Magnetic Resonance Imaging, CT: Computerized Tomography, EA: Ebstein's Anomaly, LVNC: Left Ventricular Noncompaction.
